# Effects of heat stress on egg performance in laying hens under hot and humid conditions

**DOI:** 10.14202/vetworld.2025.851-858

**Published:** 2025-04-19

**Authors:** Suchawadee Tesakul, Watcharapong Mitsuwan, Yukio Morita, Warangkana Kitpipit

**Affiliations:** 1 Akkhraratchakumari Veterinary College, Walailak University, Nakhon Si Thammarat, Thailand; 2 One Health Research Center, Walailak University, Nakhon Si Thammarat, Thailand; 3 Center of Excellence in Innovation of Essential Oil and Bioactive Compounds, Walailak University, Nakhon Si Thammarat, Thailand; 4 Department of Veterinary Medicine, School of Veterinary Medicine, Azabu University, Kanagawa, Japan; 5 Food Technology and Innovation Center of Excellence, Walailak University, Nakhon Si Thammarat, Thailand

**Keywords:** egg production, heat stress, hot and humid conditions, laying hens

## Abstract

**Background and Aim:**

Egg production is a critical indicator of reproductive efficiency in laying hens. High environmental temperatures and humidity expose laying hens to heat stress, adversely affecting egg production, egg quality, feed intake, blood chemistry, health, and behavior. Despite the global economic significance of egg production, research on the impact of heat stress in tropical climates, particularly in locally adapted breeds, remains limited. This study investigates the effects of heat stress on egg production in a new synthetic breed of laying hens raised under hot and humid conditions.

**Materials and Methods:**

This study utilized secondary data from the Ligor chicken development project, covering the period from January 1 to December 31, 2023. A total of 1293 daily records of egg production from 872 laying hens, aged between 20 and 70 *week*s, were analyzed. Egg production parameters, including hen day production (HDP) and average egg weight (AEW), were recorded daily. Environmental data, including temperature and humidity, were collected at hourly intervals using a data recorder. The heat stress index (HSI) was calculated based on temperature and relative humidity values. Statistical analyses, including regression modeling, were performed to assess the relationship between HSI and egg production.

**Results:**

The findings demonstrated that heat stress negatively impacted egg production. A statistically significant negative correlation was observed between HSI and both HDP and AEW (p < 0.05). For each unit increase in HSI, HDP decreased by 1.29%, and AEW declined by 0.18 g. These results indicate that heat stress adversely affects the productivity of laying hens in tropical climates.

**Conclusion:**

Heat stress significantly reduces egg production and egg weight in laying hens under hot and humid conditions. The findings highlight the need for adaptive breeding strategies and improved environmental management to mitigate the adverse effects of heat stress. Future research should focus on genetic selection for heat resistance and the integration of precision farming techniques to optimize poultry production in tropical regions.

## INTRODUCTION

The egg industry has experienced rapid growth due to increasing global demand and advancements in genetic selection, which have enhanced the productivity of specialized laying hen strains, particularly in temperate regions [1]. Poultry breeding companies have focused on optimizing genetic potential to maximize egg yield while improving feed conversion efficiency. Egg production is a key economic trait and an essential measure of reproductive efficiency in laying hens, influenced by multiple factors such as breed selection, nutrition, water intake, light intensity and duration, disease, management practices, and environmental conditions, particularly heat stress [1].

Heat stress poses a major challenge to the poultry industry, especially in tropical regions where high temperatures and humidity significantly affect egg production [2, 3]. Extreme weather conditions and persistent heat stress disrupt hen physiology, leading to reduced egg production efficiency, compromised health, and increased mortality rates [4–6]. Mashaly *et al*. [7] reported that 31-week-old laying hens exposed to prolonged heat stress exhibited significant declines in body weight, feed intake, egg production rate, and egg weight. The optimal environmental temperature for laying hens ranges between 66°F and 72°F [8]; exceeding this range necessitates thermoregulatory adjustments that can negatively impact productivity. Rising global temperatures and the increasing frequency of heatwaves further exacerbate the challenges faced by poultry farmers in maintaining optimal production conditions.

Although extensive research has been conducted on heat stress in poultry, most studies focus on laying hens bred for temperate or arid climates. Limited research has explored the impact of heat stress on hens specifically bred for tropical environments, where humidity and persistent heat stress are prevalent challenges. The adaptation and productivity of imported layer breeds in tropical climates remain underexplored, creating a gap in the understanding of their genetic potential under such conditions. Furthermore, studies on locally adapted or synthetically bred tropical layer hens are scarce, despite their potential to perform better under high heat and humidity.

Given the limited research on heat stress in tropical poultry production, this study aims to investigate the impact of heat stress on egg production, egg quality, and overall productivity in a newly developed synthetic breed of laying hens raised under hot and humid conditions. The findings will provide valuable insights for improving breeding strategies, developing heat-resistant layer breeds, and optimizing poultry management practices in tropical regions.

## MATERIALS AND METHODS

### Ethical approval

All data used in this study were obtained as secondary data from the Ligor chicken development project. Consequently, approval from the Animal Care and Use Committee was not required, as the information was sourced from an existing database and did not involve the direct use of animals in the study.

### Study period and location

The study data were collected from January 1 to December 31, 2023, as part of the “Ligor chicken development project” in Nakhon Si Thammarat, Thailand.

### Animal management, location, and housing

As this study focuses on heat stress, the housing and management practices were designed to replicate typical conditions in tropical climates, where high temperatures and humidity prevail. The laying hens were housed in floor pens using a deep litter system for a period of 72 weeks. The farm is situated in Nakhon Si Thammarat, on the southeastern coast of Thailand, a region characterized by a persistently tropical climate with high temperatures, substantial rainfall, and elevated humidity throughout the year. These climatic conditions significantly impact local agricultural practices, necessitating specific adaptations, particularly to manage heavy rainfall during the latter part of the year [9].

The poultry house was designed as an open housing system, measuring 5 m in width and 25 m in length. It was divided into four equally sized cages, each measuring 5 × 5 m. This housing structure was intended to ensure adequate air circulation, which is essential for maintaining a suitable environment for the hens, particularly given the region’s hot and humid climate.

All hens had access to clean water and a nutritionally balanced diet to support their health and productivity. Proper feed management is crucial for sustaining breeder hens, as it ensures optimal egg production through controlled feed intake and regular flock monitoring. The breeder feed, particularly formulated for breeder hens, was fortified with essential micronutrients, including trace minerals and vitamins, to enhance fertility and hatchability while maintaining peak egg production. The laying hens were fed once daily at 8 a.m., with an average feed intake of approximately 120 g/hen. During the egg production period, all birds received a diet containing 17% crude protein, 2750 Kcal ME/kg of feed, 3.5% calcium, and 0.5% available phosphorus. In addition, the hens were exposed to 14 h of light/day, as maintaining a consistent lighting schedule is critical for sustaining egg production.

### Egg collection and heat stress index (HSI) calculation

To evaluate the impact of heat stress on egg performance, daily egg production and environmental data were collected and analyzed using the HSI. The dataset comprised 1293 daily records of egg production from 872 laying hens aged between 20 and 70 weeks. Egg production was recorded 3 times/day at 8 a.m., 11 a.m., and 3 p.m., with the weighted average egg mass calculated for each replication. In this study, the traits analyzed included hen-day production (HDP) and average egg weight (AEW). HDP was calculated by dividing the total number of eggs collected by the total number of live hens per day in each group. AEW was determined by selecting a representative sample, accurately weighing each egg, recording the measurements, and computing the average. Additional factors, such as the age of the laying hens and cage position, were also recorded for model adjustments. Hens were monitored daily, and the weight of deceased birds was considered to adjust daily feed consumption calculations.

Meteorological data were collected using a data logger (Elitech GSP-6, Elitech, USA) to measure temperature and relative humidity throughout the study period. The device was placed inside the poultry house, and temperature and humidity levels were recorded at hourly intervals. The study area’s environmental conditions were characterized by consistently high temperatures and humidity throughout the year.

**Figure 1:**
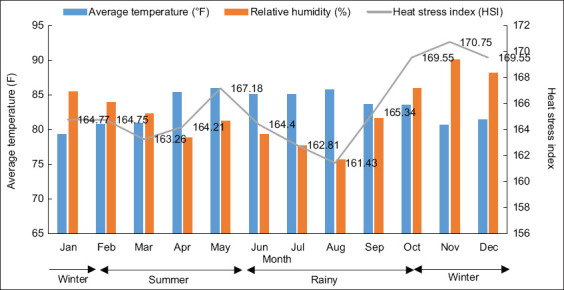
Climate conditions: average air temperature, relative humidity, and heat stress index values from January 2023 to December 2023.

The weather in the study region is classified into three seasons: (1) Summer (February to May), (2) Rainy season (June to October), and (3) Winter (November to January) [9]. Temperature variations ranged from 79.30°F to 85.92°F, with the lowest average temperature recorded in January (79.30 ± 1.87°F) and the highest in May (85.92 ± 1.76°F). Relative humidity peaked in November (90.07 ± 3.92%) and was lowest in August (75.65 ± 4.20%), as illustrated in Figure 1.

The HSI was calculated based on two parameters: Temperature and relative humidity, as described by Phongpipat *et al*. [10] and Hy-Line International [11] in the following equation.

HSI = Dry bulb temperature (°F) + Relative humidity (%)

### Statistical analysis

The data were checked for normality using the Shapiro–Wilk test, for homogeneity of variance using Levene’s test, and outlier data were removed before analysis. Statistical analysis was conducted using the General Linear Model Procedure of SAS OnDemand for Academics [12]. All statistically significant differences were based on p < 0.05 unless noted otherwise. All data were processed and categorized using Microsoft Excel. The relationship between HSI and egg performance was determined using the linear regression method. The statistical equation model used in this study is as follows:

y_ijk_ = μ + collection date_i_ + flock_j_ + b1(age)_ijk +_ b2(HSI)_ijk_ + e_ijk_

Where: y_ijk_ is the individual observation of consideration traits; μ is overall means; collection date_i_ is the collection date effect, flock_j_ is the flock effect; (age)_ijk_ is age of laying hens; (HSI)_ijk_ is the heat stress index value; b1 and b2 are regression coefficients; e_ijk_ is residual error NID~(0,).

Furthermore, regression analysis was used to generate the statistical equation model for future prediction as follows:

y_i_ = a + b(HSI_i_)

Where y_i_ is the individual observation of consideration traits; a is the y-intercept; b is regression coefficients; HSI_i_ = Heat stress index values.

## RESULTS AND DISCUSSION

### Climatic conditions during the study period

The study was conducted from January to December 2023, covering all seasons in Nakhon Si Thammarat, Thailand. As illustrated in Figure 1, the province experiences consistently high temperatures and humidity throughout the year, which is characteristic of a tropical monsoon climate. The average air temperature was 83.41°F (28.56°C), with the highest temperatures recorded in May and the lowest in November. Relative humidity remained consistently high, averaging 82.53% annually. The persistently elevated temperatures and humidity levels during the study period emphasize the potential risk of heat stress impacting egg production in laying hens. These climatic conditions are comparable to those in other tropical monsoon regions, including South and Southeast Asia, West Africa, and Central and South America. Mashaly *et al*. [7] reported that temperatures exceeding 86°F (30°C) significantly impair egg production and feed efficiency in laying hens. Consequently, effective climate management is crucial for ensuring livestock health and production sustainability in the region [13].

Thailand’s climate is predominantly influenced by two seasonal monsoons. The Southwest Monsoon (May–October) carries humid air from the Andaman Sea and the Gulf of Thailand, contributing to low-pressure systems over China (Tibetan Low) [14]. In contrast, the Northeast Monsoon (November–February) brings heavy rainfall to the east coast, particularly affecting Surat Thani, Nakhon Si Thammarat, and Songkhla due to moisture from the Gulf of Thailand [9].

The annual average HSI in the region was 165.67. The highest HSI value (170.75) was recorded in November, indicating a period of severe heat stress, while the lowest value, 161.43, occurred in August. HSI values exceeding 160 are generally associated with heat stress and can significantly affect livestock performance [10, 11]. In addition, Kilic and Ercan [3] reported that elevated indoor temperature and humidity levels contribute to heat stress in poultry.

Numerous studies have reported climate data from various countries, highlighting variations in temperature and humidity across tropical and sub-tropical regions. In Thailand, Loengbudnark *et al*. [15] found that Khon Kaen Province, located in the northeast, has an average temperature of 26.9°C, with the highest recorded temperature in April and the lowest in January. Relative humidity remains above 67%, resulting in an average temperature-humidity index (THI) of 76.6, peaking at 82 from April to July and decreasing to 68 in January and December. Similarly, in Sri Lanka, temperatures range between 28.0°C and 32.0°C, with THI values fluctuating from 68.57 to 83.17, reflecting regional differences in humidity and heat stress levels [16].

In India’s tropical region of Palakkad, maximum daytime temperatures vary from 32.27°C to 41.05°C, while relative humidity ranges between 44.1% and 92.3%, leading to THI values between 71.88 and 83.21 from January to March [17]. Closer to the equator, in Cuba, significant diurnal THI variations occur, starting at 69.59 in the morning, increasing to 79.61 at midday, and peaking at 91.69 in the afternoon due to rising daytime temperatures and humidity [18]. Conversely, Tanzania experiences considerable climate variability, with THI values ranging from 61 to 86 across different regions of the country [19].

These fluctuations in temperature and humidity highlight the impact of geographic location, seasonal patterns, and local environmental conditions. Understanding these climatic variations is essential for developing effective heat stress management strategies, particularly in agriculture and livestock production. By implementing appropriate measures to mitigate heat stress, farmers can enhance animal welfare, improve productivity, and support the long-term sustainability of food production systems.

### Egg performance

The descriptive statistics of egg performance obtained during the study period are presented in Table 1. The average age of laying was 43.82 ± 13.24 weeks. The average HDP and AEW were 55.72 ± 14.38% and 57.93 ± 3.38 g/egg, respectively. HDP ranged from 5.92% to 88.89% and egg weight ranged from 40 g to 69.42 g/egg.

**Table 1:** Descriptive statistics of egg performance.

Traits	Average ± SD	Min	Max
Age of laying hen (weeks)	43.82 ± 13.24	20.00	70.00
Hen day production (%)	55.72 ± 14.38	5.92	88.89
Egg weight (g/egg)	57.93 ± 3.38	40.00	69.42

SD=Standard deviation, Min=Minimum, Max=Maximum

**Figure 2:**
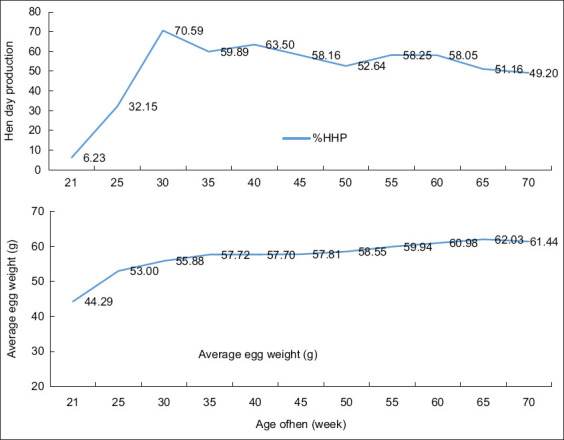
A typical production curve for a laying flock. (a) Hen day production and (b) Average egg weight.

The observed trends in egg production and weight are consistent with previous studies by Ribeiro *et al*. [8], Narinc *et al*. [20], and Star *et al*. [21] and likely reflect the influence of the region’s tropical climate, particularly the effects of heat stress. Figure 2 illustrates a typical egg production curve showing changes in egg production and weight. During the study period, egg production increased sharply, peaked at approximately 70.59% around the 10^th^ week, and gradually declined to approximately 49% by the 40^th^ week of lay. This result aligns with previous studies by Saeed *et al*. [4], Narinc *et al*. [20], Star *et al*. [21] and Ezieshi *et al*. [22], reinforcing the established pattern of egg production in poultry. Similar trends have been observed in White Leghorn pullets, where egg weight increased during the first 1–5 weeks of production, followed by a linear decrease as production peaked [23].

However, the higher egg production observed in chickens raised in closed housing compared to those raised in open systems can be attributed to improved environmental control, better nutrition, and enhanced management practices. Closed housing systems provide optimal conditions by regulating temperature, humidity, and lighting, which help reduce stress and promote consistent egg production [24]. In addition, these systems minimize exposure to external factors, such as predators, diseases, and harsh weather conditions, further improving the overall flock performance. In contrast, chickens raised in an open system experience greater environmental fluctuations and potential health challenges, which can negatively impact their egg-laying capacity.

### Relationship between HSI and egg performance

**Figure 3:**
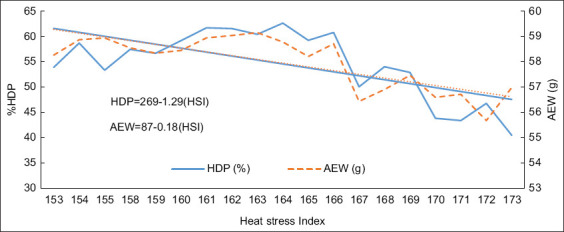
Relationship between heat stress index and hen day product (HDP) and average egg weight (AEW) in laying hens under hot and humid conditions.

The HSI and egg production relationship exhibited a negative trend (Figure 3). Specifically, for every unit increase in HSI, HDP decreased by 1.29%, and AEW declined by 0.18 g. These findings are consistent with previous studies by Kilic and Ercan [3], Saeed *et al*. [4], Mashaly *et al*. [7], Weerasinghe *et al*. [16], Star *et al*. [21], Gençoğlan [25] and Bekele [26], which reported significant declines in egg production and quality under heat stress conditions. For instance, Deng *et al*. [27] observed a 28.8% decline in egg production following a 12-day heat stress period, whereas Star *et al*. [21] documented reductions of 36.4% in egg production and 3.41% in egg weight. In addition, Mashaly *et al*. [7] reported notable decreases in egg production (28.8%), feed intake (34.7%), and body weight (19.3%) in hens exposed to prolonged heat stress for 5 weeks. The variability in these effects can be attributed to factors such as age, sex, breed, genetic background, and the intensity and duration of heat stress exposure [15, 18].

The decreases in egg production and feed intake can be physiologically explained by the disruptive effects of heat stress on hormonal regulation. High temperatures impair the secretion of gonadotropin-releasing hormone from the hypothalamus, leading to decreased levels of luteinizing hormone and follicle-stimulating hormone. This hormonal imbalance hinders follicular maturation and ovulation, ultimately reducing egg production. Previous studies by Hy-Line International [11], Vandana *et al*. [24], Bekele [26], Yan *et al*. [28], Yalcin *et al*. [29] and Lara and Rostagno [30] have confirmed that heat stress disrupts the hypothalamic-pituitary-gonadal axis, altering the synthesis and secretion of reproductive hormones. These disruptions further contribute to declining egg production and reproductive efficiency in laying hens.

Understanding these physiological mechanisms is essential for developing effective interventions to mitigate heat stress and maintain optimal laying performance. Strategies such as genetic selection, cooling systems, and nutritional adjustments can address both environmental factors and hormonal regulation, improving the resilience of laying hens to heat stress. For example, Javandel Soum Sarai *et al*. [31] suggested that supplemental chromium picolinate can effectively alleviate the adverse effects of heat stress, enhancing both growth performance and immune function in broilers.

Moreover, HSI values remained consistently elevated throughout the study period, exceeding the 166-a threshold typically associated with reduced flock performance [11]. Despite these challenging conditions, the hens demonstrated greater heat tolerance and continued to produce eggs at an adequate yield, although below their genetic potential, as observed in their original herd. This suggests that layer hens may have developed adaptive mechanisms to mitigate the adverse effects of heat stress. Understanding and controlling environmental conditions are critical for sustainable poultry production and animal welfare [30,32].

Therefore, mitigation strategies such as genetic selection for heat tolerance and improved housing designs are essential to reduce heat stress. For instance, Tohidi *et al*. [33] investigated the expression of heat shock protein (HSP) genes in Khorasan native chickens under acute heat stress (42°C, 50% humidity). The results of the present study suggest that Khorasan native chickens exhibit strong heat resistance, with HSPA2 contributing to protein stability under high temperatures. In addition, Oluwagbenga and Fraley [32] emphasized the importance of minimizing stress through optimized housing, nutrition, and management strategies to enhance poultry welfare and productivity.

In the future, poultry farmers must adopt climate-resilient practices to mitigate the risks associated with heat stress. This includes closely monitoring the temperature and humidity levels to optimize the housing conditions. During the daytime, when temperatures rise and relative humidity drops, evaporative cooling methods such as foggers or cooling pads are effective. However, foggers may intensify heat stress at night, when temperatures decrease and humidity rises. In high-humidity conditions, increased air movement through fans can provide relief by creating a wind chill effect, which reduces the perceived temperature of birds [34].

## CONCLUSION

This study provides novel insights into the impact of heat stress on egg production in laying hens raised under hot and humid conditions, focusing on a newly developed synthetic breed specifically adapted to tropical climates. The findings establish a significant negative correlation between the HSI and key production parameters, with each unit increase in HSI resulting in a 1.29% decline in HDP and a 0.18 g reduction in AEW (p < 0.05). These results confirm that heat stress is a major constraint on poultry production in tropical regions, reinforcing the need for targeted breeding and management strategies to mitigate its adverse effects.

The originality of this study lies in its extensive dataset, which includes 1293 daily records from 872 laying hens over a complete production cycle under real-world tropical conditions. Unlike previous studies that have primarily focused on commercial layer breeds in controlled environments, this research uniquely examines a locally developed synthetic breed, offering new perspectives on genetic adaptability and production efficiency in heat-stressed environments. The study’s robust statistical approach further strengthens its conclusions, providing a reliable foundation for future research and industry applications.

Despite its strengths, the study has some limitations. The reliance on secondary data constrained the ability to control microclimatic variations within the housing system. In addition, physiological stress markers, such as corticosterone levels or oxidative stress indicators, were not included, which could have provided deeper insights into the biological responses of hens to heat stress.

Future research should explore genetic and nutritional interventions to enhance heat resilience in laying hens. Investigating molecular and physiological markers of heat tolerance could facilitate selective breeding programs to improve adaptation to tropical climates. In addition, evaluating the effectiveness of housing modifications, cooling systems, and precision farming technologies – such as automated climate monitoring and feed optimization – could provide practical solutions for sustaining egg production under extreme environmental conditions.

By integrating genetic, nutritional, and environmental innovations, the poultry industry can develop resilient layer breeds capable of maintaining optimal productivity in hot and humid climates. This study contributes to the growing body of knowledge on heat stress in poultry and serves as a foundation for future advancements in sustainable egg production under tropical conditions.

## AUTHORS’ CONTRIBUTIONS

WK: Conceptualized, experimental design, analyzed and interpreted the data, and drafted the manuscript. ST: Data collection, analysis, and inter-pretation and drafted and reviewed the manuscript. YM: Data collection and reviewed and edited the manuscript. WM: Validation and reviewed and edited the manuscript. All authors have read and approved the final manuscript.
